# The Influence of Silicon Content and Synthesis Atmosphere on the Electrical Properties and Chemical Composition of Ru–Si–O Nanocomposites

**DOI:** 10.3390/molecules31111802

**Published:** 2026-05-24

**Authors:** Aleksandra Wilczyńska, Aleksandra Wójcicka, Andrzej Taube, Mateusz Łakomski, Tomasz N. Kołtunowicz

**Affiliations:** 1 Department of Electronics and Information Technology, Lublin University of Technology, Nadbystrzycka 38A, 20-618 Lublin, Poland; 2 Łukasiewicz Research Network—Institute of Microelectronics and Photonics, Lotnikow Ave 32/46, 02-668 Warsaw, Poland; aleksandra.wojcicka@imif.lukasiewicz.gov.pl (A.W.); andrzej.taube@imif.lukasiewicz.gov.pl (A.T.); 3 Department of Semiconductor and Optoelectronic Devices, Lodz University of Technology, Politechniki Ave 8, 93-590 Lodz, Poland; mateusz.lakomski@p.lodz.pl; 4 Department of Electrical Devices and High Voltage Technology, Lublin University of Technology, Nadbystrzycka 38A, 20-618 Lublin, Poland; t.koltunowicz@pollub.pl

**Keywords:** Ru-Si-O nanocomposites, magnetron sputtering, impedance spectroscopy, dielectric properties, oxygen content, electron tunneling

## Abstract

This paper presents the results of the preparation and electrical characterization of Ru–Si–O thin-film nanocomposites deposited by magnetron sputtering (pDC) with varying oxygen content ranging from 0% to 50%. Measurements were conducted over a wide frequency range of 50 Hz–5 MHz and temperatures of 20–373 K. Conductivity analysis revealed that DC conduction occurs at low frequencies (≤10^3^ Hz), while an increase in conductivity associated with electron tunneling mechanisms is observed at higher frequencies. The determined charge transport activation energies range from 3 × 10^−4^ eV for the oxygen-free sample to 6 × 10^−2^ eV for the high-oxygen samples, indicating a significant effect of composition on the conduction mechanisms. In samples containing 30% and 50% oxygen, two characteristic frequency ranges for the activation of transport processes were observed (e.g., ~10^2^–10^3^ Hz and 10^4^–10^6^ Hz), suggesting the coexistence of multiple tunneling mechanisms. Phase angle analysis revealed a transition from values near –90° at 151 K to values near 0° at 333 K, characteristic of parallel RC systems. The minimum dielectric loss tangent occurs in the range of 10^3^–10^5^ Hz, corresponding to Maxwell–Wagner relaxation. The dispersion coefficient α reaches maximums in two frequency ranges, decreasing with increasing oxygen content. EDS analysis showed a decrease in Ru content from ~24.9 at.% (0% O_2_) to ~0.7 at.% (50% O_2_) and an increase in oxygen content to ~78 at.% at 10% O_2_. The results confirm the transition from metallic conduction to tunneling and hopping mechanisms with increasing oxidation state of the structure.

## 1. Introduction

The miniaturization of electronic devices, a consequence of Moore’s law, has driven the development of the semiconductor industry for over fifty years [1,2]. Reducing the size of transistors allows for more components to be placed on a chip, increasing computing power while reducing energy consumption and cost [3]. Modern devices are characterized by high integration and functional complexity. Nanocomposites are a key element of semiconductor technologies, enabling miniaturization at the nanometer scale [4,5]. Atomically thick thin films are essential in integrated circuits, displays, sensors, and photovoltaic cells [6]. Thin-film technology allows for precise control of their physical and electrical properties [7,8]. In MEMS microsystems, thin semiconductor films increase efficiency, miniaturization, and cost-effectiveness by enabling the integration of materials with diverse properties [9,10].

Magnetron sputtering is the dominant PVD technique for producing thin functional layers. It deposits metals, alloys, or oxides on a substrate in a vacuum process, allowing for control of particle size through process parameters [11,12].

Accurate structural and morphological analysis of magnetron layers is crucial to understanding their functional properties. Energy-dispersive X-ray spectroscopy (EDS) enables the study of phase composition, elemental distribution, and morphology for metals, alloys, ceramics, and polymers [13,14].

Ruthenia and its oxides (especially RuO_2_) have attracted attention due to their unique electrical and chemical properties, which hold promise in electrochemistry, electromaterials, and thin-film dielectric devices [15,16,17,18]. Doping SiO_2_ enables control of the micro/nanostructure and conductivity from metallic to insulating functions. Reactive magnetron sputtering allows modification of oxidation state, composition, and morphology, influencing dielectric properties and conductivity [19,20,21].

Ru–Si–O structures are used in thin-film materials for electronics, sensors, and catalysis, serving both conductive and insulating functions. Their properties can be controlled by the oxygen content during deposition, which allows for the tuning of electrical parameters. They are characterized by electrical stability and resistance to high temperatures, making them promising for applications such as Schottky contacts and capacitors [22,23]. They also exhibit good catalytic properties, and the addition of silicon increases the durability of catalysts [24,25,26].

The conductivity and magnetoresistance of these layers are strongly temperature-dependent, which influences electron transport mechanisms and the variability of electrical properties [27,28]. This is crucial for the design of reliable microelectronic systems [29,30,31]. Ru–Si–O structures can also be used in humidity sensors [19,32] and devices that utilize magnetic field control of charge transport, such as memories or adjustable resistance elements [33,34].

In medicine and bioengineering, Ru-Si-O nanocomposites are mainly used as electrode materials in biosensors, elements of field-effect transistors (Bio-FETs) and intelligent carriers in imaging diagnostics [35,36,37]. Ruthenium and its oxides have significantly better biocompatibility than copper used in electronics. The addition of ruthenium in Cu-Ru electrodes allows copper to maintain its good conductivity while eliminating its toxicity to cells. Such electrodes are used in impedance sensors that distinguish between living and dead cells. Their operation is based on changes in the AC impedance at the cell surface: their membranes, acting as insulators with a specific capacitance, affect the electrical parameters of the system, which can be described using equivalent models [38]. RuO_2_ nanoparticles exhibit high electrocatalytic activity, making them suitable for use in amperometric sensors. They enable direct monitoring of H_2_O_2_ secretion by cancer cells, which is crucial for assessing oxidative stress and early diagnosis of disease [37].

Ru-Si-O nanocomposites are important in environmental protection, including gas monitoring, water treatment, and energy production. Their high selectivity and moisture resistance make them suitable for detecting CO, NO_x_, and alcohol vapors. In SnO_2_-based sensors, the addition of SiO_2_ and RuO_2_ improves the material’s surface and electrical properties. Thanks to its optoelectronic properties, the Ru-Si-O system is a useful component of photocatalytic systems. In heterojunctions such as RuO_2_/TiO_2_, ruthenium acts as a cocatalyst, effectively splitting electron–hole pairs generated by light [22,39,40,41]. The electrochemical stability of RuO_2_ allows it to be used in pH sensors intended for use in precision agriculture and groundwater monitoring [22].

The highest sensitivity of resistive sensors is achieved near the percolation threshold. In this state, the material is at the boundary between insulator and conductor. Gas adsorption or biopolymer attachment changes the local electrostatic potential, which can block or enable tunneling current conduction. Capacitive sensors exploit the strong increase in dielectric permittivity, which is highly sensitive to the distances between conductive grains. Mechanical changes, such as stress or polymer swelling, cause noticeable changes in capacitance, enabling the creation of highly sensitive strain sensors [42,43].

## 2. Materials and Methods

### 2.1. Deposition of Ru-Si-O Nanocomposite Films

Ru–Si–O thin films with a thickness of 100 nm were deposited on quartz substrates using a Leybold Z400 (Leybold, Germany) sputtering system in pDC mode, with a base pressure of 10^−6^ mbar, according to related works [44]. The processes were carried out at room temperature using a 3-inch RuSi target with a nominal composition of 50:50 at.% in an O_2_/Ar gas mixture, where high-purity (6N) oxygen content varied from 0 to 50% in steps of 10%. The target-to-substrate distance was approximately equal to 3 cm, while the total working pressure during sputtering was maintained at the level of 10^−2^ mbar. More information about Ru-Si-O layer deposition and characterization can be found in work [45]. The silicon substrates had a resistivity of about 10 Ω·cm, which is sufficiently high to ensure that the substrate contribution to the AC measurements of the investigated nanocomposites is negligible.

### 2.2. Measurements of AC Properties of Ru-Si-O Nanocomposites Using Impedance Spectroscopy

Impedance spectroscopy is an analytical technique used to characterize the electrical and structural properties of nanocomposites. This method involves applying a small, alternating electrical signal at a variable frequency and measuring the material’s current response. In this way, the analysis of phenomena occurring within the nanocomposite structure at the nanometric level, grain boundaries, and interfaces can be done [46]. Impedance spectroscopy allows for the identification of dominant conduction mechanisms in nanocomposites, such as delocalized carrier transport, electron tunneling between nanoparticles, and variable-range hopping [47]. In metal–insulator nanocomposites, it is possible to distinguish contributions from metallic grains, grain boundaries, and interfaces [48,49]. One of the key applications of impedance spectroscopy is determining the percolation threshold in nanocomposites containing conductive nanoparticles in a nonconducting matrix. The percolation threshold is the critical conductive concentration at which a continuous network is formed, allowing free current flow. Below this threshold, nanocomposites exhibit almost purely capacitive behavior, while above this threshold, resistive conductivity dominates [50,51]. In nanocomposites, where nanoparticles are separated by thin dielectric layers, conduction can occur via quantum tunneling of electrons. Impedance spectroscopy allows us to distinguish between conduction via direct contact of nanoparticles and conduction by tunneling, which is crucial for understanding the conduction mechanism in the structure.

According to Mott’s model tunneling theory, this phenomenon can occur in two ways. The first form of tunneling occurs when electrons are poorly localized and at temperatures close to or lower than the temperature of liquid helium, when tunneling occurs via a variable-range hopping mechanism. This process results from the fact that the thermal energy is insufficient to allow the electron to tunnel to the nearest potential well. Consequently, the electron travels a longer distance to locate a well characterized by a suitably low energy level. This leads to a small temperature dependence of conductivity [52]. Conductivity in this type of phenomenon is described by Equation (1):

(1) $$\sigma \sim exp\left[-{\left(\frac{T}{T_{0}}\right)}^{-0.25}\right]$$

where σ—conductivity, *T*—temperature, *T*_0_—density of states at the Fermi level.

The second mechanism occurs under conditions of strong electron localization and at elevated temperatures. In these circumstances, electrons tunnel between adjacent potential well [52]. The key parameter determining conductivity is the electron hopping probability p per unit time. In this case, the mechanism is described by Equation (2), and the electron hopping probability.

(2) $$\sigma \sim exp\left(-2\cdot \alpha \cdot r-\frac{\Delta E}{k\cdot T}\right)$$

where −2∙α∙r—the rate of fall of the electron wave function, *∆E*—single electron hopping activation energy, *k*—Boltzmann’s constant, *T*—temperature.

**Figure 1:**
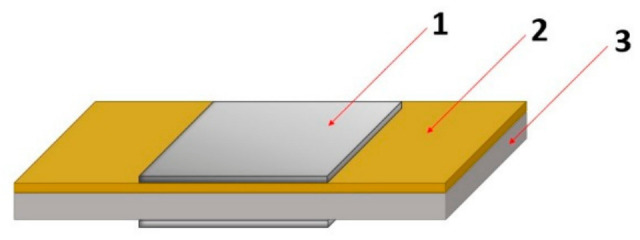
Cross-section of Ru-Si-O nanocomposite sample: 1—silver contact, 2—nanocomposite layer, 3—silicon substrate.

The structures studied are referred, according to the literature, as mictamic alloys, which constitute unique nanocomposite material systems. The Ru-Si-O mictamic alloy is defined as a mixture of two immiscible binary oxides, RuO_x_ and SiO_2_, with different crystal structures, leading to microstructural amorphization. In this structure, metallic ruthenium Ru nanoparticles are surrounded by an RuO_x_ oxide shell, and the entire structure is embedded in an amorphous silicon dioxide SiO_2_ matrix [53]. Characterization of electrical properties, including resistance Rp, capacitance Cp, phase shift angle θ, and dielectric loss tangent tan δ, was performed in the frequency spectrum ranging from 50 Hz to 5 MHz and the temperature range of 20–373 K. The measurements were performed using measurement equipment consisting of a Hioki 3536 RLC meter (Hioki, Japan), a helium cryostat, a temperature controller, and a vacuum pump to ensure low temperatures and eliminate condensation in the measurement system. This paper presents results for selected temperatures. Figure 1 illustrates the structure of the tested structures, each 100 nm thick, on which silver paste electrodes with a surface area of 2 × 2 mm were deposited.

### 2.3. Characterization of the Chemical Composition of Nanocomposites Using the EDS Method

The chemical composition was examined using an energy-dispersive X-ray spectroscopy (EDS) system (EDAX Octane Elite, APEX™ EDS, EDAX LLC, Tokyo, Japan) working with a scanning electron microscope SEM. The measurements were performed at an accelerating voltage of 20 kV using a secondary electron detector. EDS analysis was conducted to confirm the elemental composition of the Ru–Si–O nanocomposite, with the same accelerating voltage, carefully selected to ensure adequate excitation of Ru, Si and O peaks. The samples of nanocomposites were prepared on the silicon wafer, which has influence on the signal overlap. Simultaneously, the operating parameters as: 0.5 nA beam current, deadtime DT, counts number and the working distance were kept at the same optimal level for all tested samples.

## 3. Result

### 3.1. Measurements of Electrical Parameters of Ru–Si–O

**Figure 2:**
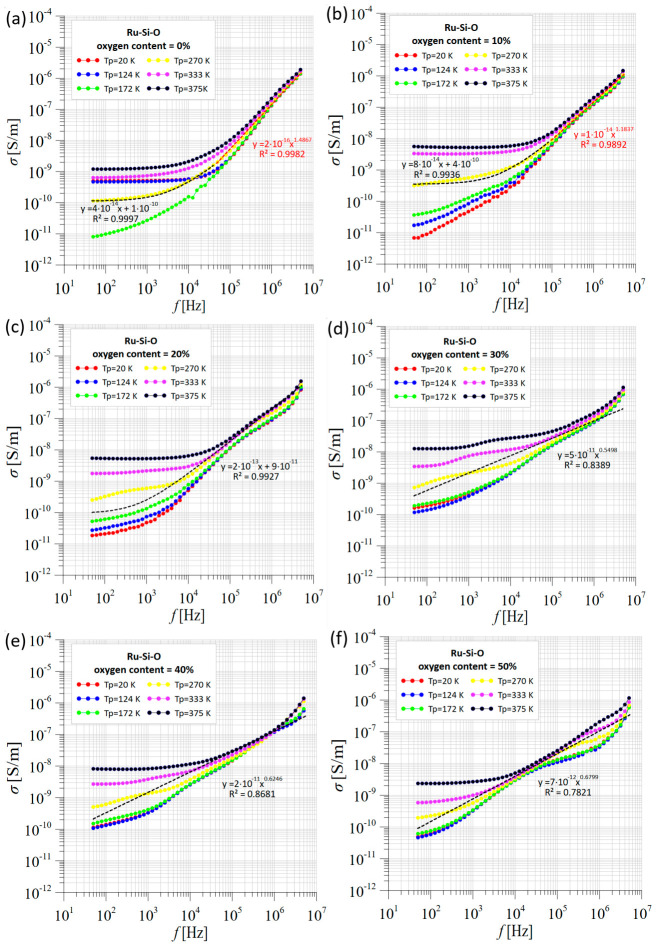
Dependence of conductivity on the frequency of structures with a trend line at selected measurement temperatures obtained in an oxygen atmosphere with the following contents: (**a**)-0%, (**b**)-10%, (**c**)-20%, (**d**)-30%, (**e**)-40%, (**f**)-50%.

Resistance, capacitance, phase shift angle, and dielectric loss tangent were measured using the four-point method over a frequency range of 50 Hz to 5 MHz and a temperature range of 20 K to 373 K. The description of the stand and the measurement method is presented in the articles [54]. The parameters were determined using a linear array of four collinear wires, two connected to each contact (L_CUR_, L_POT_ and H_CUR_, H_POT_), placed on both sides of the sample, which was measured in a current perpendicular to plane configuration. Square silver paste contacts with an area of 4 mm^2^ were painted on the sample. Based on the obtained resistance values, the electrical conductivity of the tested samples was calculated. Figure 2 shows the dependence of conductivity on frequency for structures with different oxygen content, which allows for the analysis of charge transport properties in the tested material. Ru–Si–O layers were obtained in a single process on a large surface of a silicon substrate. For measurements, the sample was divided into smaller fragments, and measurements taken at 10 different locations showed comparable results and good layer uniformity.

Based on Arrhenius-type plots depicting the dependence of specific conductivity on the reciprocal of temperature (1/T), the activation energies of charge transport processes in the studied structures were determined (Table 1). The physical significance of positive activation energies in our nanocomposite stems from the dominant conduction mechanisms, such as defect-assisted tunneling through the oxide shells surrounding the metallic particles, a transition between tunneling, and a thermally determined hopping conduction mechanism. To justify the methodology, the activation energy was determined in the frequency range where the conductivity versus frequency relationship begins to increase with frequency. Trend lines added for dependances of conductivity in function of frequency for room temperature measurements.

**Figure 3:**
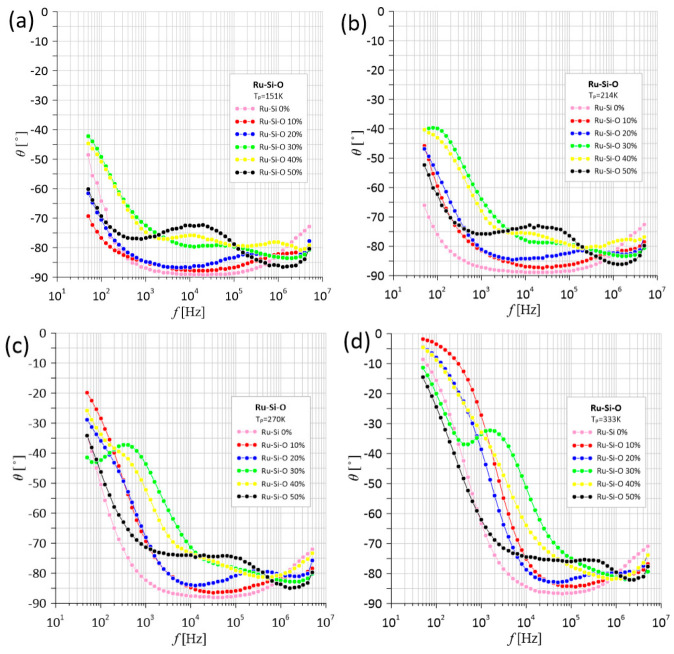
Dependence of the phase shift angle on the frequency at temperatures (**a**)-151 K, (**b**)-214 K, (**c**)-270 K, (**d**)-333 K for structures obtained at different oxygen contents.

Figure 3 shows the dependence of the phase angle on frequency for selected temperatures, 151 K, 214 K, 270 K, and 333 K, recorded for structures with different oxygen contents. Analysis of the presented characteristics allows for a detailed understanding of the changes in the electrical response of the tested materials depending on temperature and the oxygen content in the structure.

**Figure 4:**
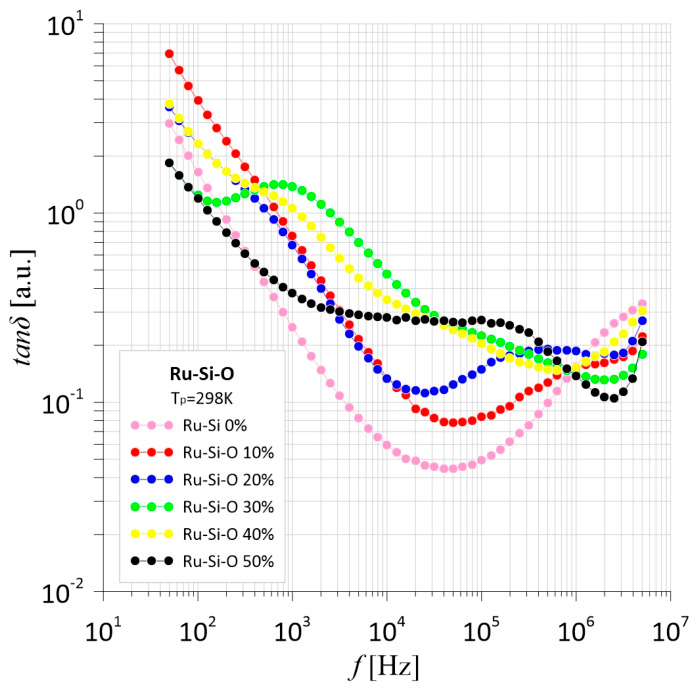
Dependence of the dielectric loss angle tangent as a function of frequency for samples obtained with different oxygen contents, measured at room temperature.

Figure 4 shows the dependence of the dielectric loss tangent as a function of frequency for samples obtained with different oxygen contents measured at room temperature. In oxide composites, the characteristic dielectric loss tangent (tanδ) as a function of frequency, visible in the graph, describes the relaxation mechanisms occurring in the material.

**Figure 5:**
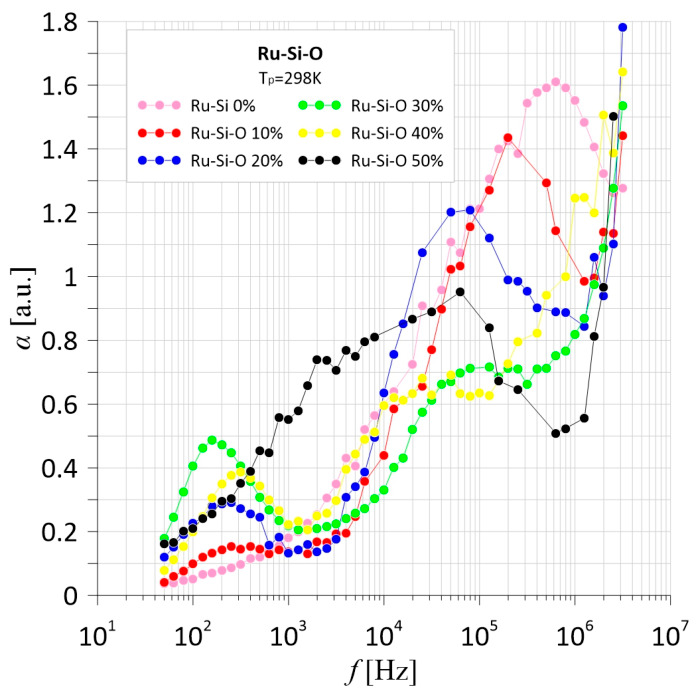
Dependence of the frequency coefficient as a function of circular frequency for samples obtained at different oxygen contents, measured at room temperature.

Figure 5 shows the frequency coefficient alpha, which in the literature is typically interpreted as a dispersion parameter describing nonlinearity in the energy distribution, for example, related to dielectric conductivity, AC dispersion, or broadly defined dielectric relaxation. This relationship is crucial in oxide materials, where structural irregularities and changes in oxygen content influence the transport and relaxation properties of the materials. The value of the frequency coefficient was determined based on Equation (4) which is closely related to Jonscher’s law.

**Figure 6:**
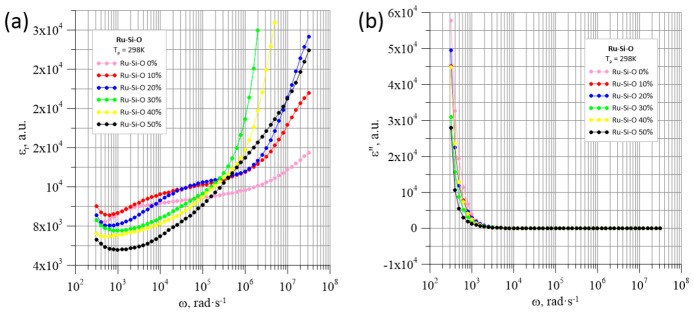
Dependence of the real part of the permittivity (**a**) and the imaginary part of the permittivity (**b**) as a function of frequency for samples obtained at different oxygen contents, measured at room temperature.

Figure 6 shows the changes in the real value ε_r_ (a) and imaginary value ε″ (b) of the dielectric permittivity as a function of circular frequency. It can be seen that εr increases with increasing temperature and frequency, and additionally, two characteristic maxima appear in the frequency spectrum, where the loss tangent reaches minima.

### 3.2. Chemical Studies of Ru-Si-O Nanocomposite

**Figure 7:**
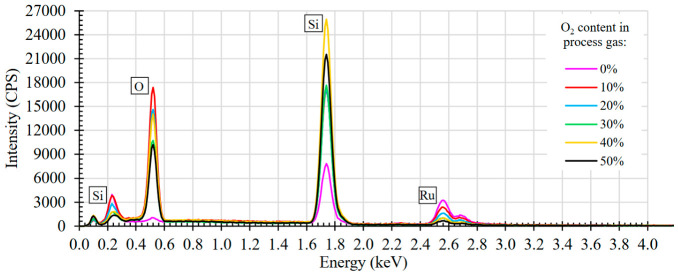
EDS spectrum of Ru-Si-O thin films for different O_2_ content in process gas.

**Figure 8:**
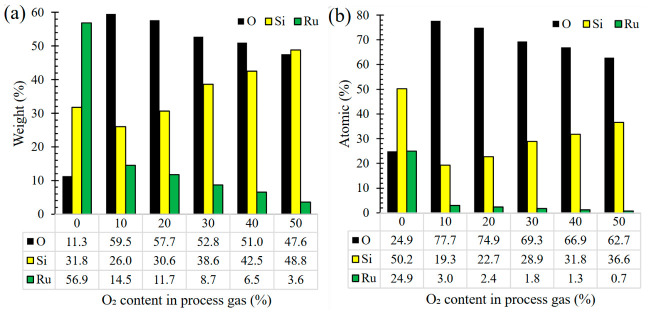
Elemental composition of Ru–Si–O thin films deposited at different oxygen concentrations: (**a**) elemental weight percentages and (**b**) atomic percentages obtained from EDS analysis.

The compositional trends obtained experimentally by EDS analysis are in line with the theoretical predictions for reactive magnetron sputtering of Ru–Si in Ar/O_2_ atmospheres [55]. It should be noted that EDS was used here as a comparative, trend-based analytical tool, rather than for absolute quantification of light elements or for direct phase and oxidation-state identification. The energy-dispersive X-ray spectroscopy (EDS) spectrum of Ru-Si-O thin films is shown in Figure 7. Figure 8 shows the elemental composition of Ru–Si–O thin layers deposited at different oxygen concentrations.

**Figure 9:**
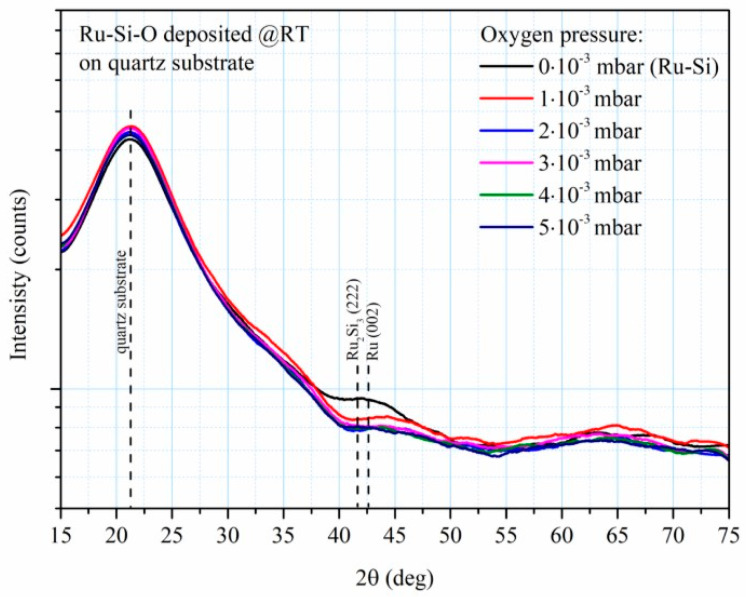
XRD pattern of Ru-Si-O thin films deposited under different oxygen pressures.

To determine the crystalline structure, X-ray diffraction was performed using a Cu lamp and a Johansson monochromator on a Empyrean system (PANalytical, Netherlands). The diffractograms are shown in Figure 9.

## 4. Discussion

### 4.1. Analysis of the Conduction Mechanism in Ru-Si-O Nanocomposites

In all tested samples, a lack of a clear frequency dependence of conductivity values was observed in the low-frequency range, indicating the dominance of static processes and limited charge carrier mobility in this temperature region. With increasing frequency and temperature, a linear increase in conductivity occurs, which is characteristic of the electron tunneling mechanism in the tested material. This behavior suggests that at higher temperatures and frequencies, additional charge transport channels are activated, associated with the quantum penetration of electrons through potential barriers between neutral potential wells or structural grains. Furthermore, in all samples, conductivity values were found to increase with increasing temperature, which is typical for materials in which conductivity is called hopping charge transfer, determined by a thermally activated charge transport mechanism. This indicates that thermal energy facilitates the hopping of electrons between localized states, leading to an increase in the overall electrical conductivity of the sample. The exception is the sample with 0% oxygen content, where a decrease in conductivity was observed up to a temperature of approximately 172 K. This behavior is characteristic of metals and corresponds to resistive conductivity, where an increase in temperature causes increased electron scattering and, consequently, a decrease in conductivity. The interpretation is purely speculative. The observed change in conductivity behavior could equally well result from alternative, physically justifiable mechanisms, such as crossover between different transport regimes, percolation effects in the environment of metallic nanograins, or intensification of electron localization in surface defect states, which does not require a change in the global electronic structure. Only above this temperature does a change in the nature of conductivity occur: conductivity begins to increase with temperature, which may indicate a transition from a metallic to a dielectric conduction mechanism. This change suggests that in this temperature range, a significant reorganization of the electronic structure or a change in the local oxidation state occurs, leading to the dominance of tunneling and hopping charge transport processes. The observed phenomenon may be due to the fact that the absence of oxygen during the sample preparation process prevented the formation of a dielectric layer in the form of SiO_2_. Consequently, the material’s structure did not form insulating oxide barriers, which in typical metal–dielectric nanocomposites play a significant role in limiting charge flow and determine the mechanism of electron tunneling. In the case of the oxygen-free sample, electrons can move directly through the silicon Si phase, leading to significantly higher conductivity at low temperatures and unusual conductivity behavior as a function of temperature and frequency. The absence of the SiO_2_ layer causes the material to exhibit a more metallic conductivity, especially at low temperatures, and the observed decrease in conductivity to approximately 172 K may be due to the dominance of electron scattering in the silicon. In the structures obtained at 30% and 50% oxygen content, the presence of more than one electron tunneling mechanism, characterized by different activation energies, was observed. This indicates that charge transport in these materials is not determined by a single, dominant process but rather results from the overlap of several parallel conduction mechanisms occurring at different frequency and temperature ranges. In the structure presented in Figure 2d, an increase in conductivity is noticeable at frequencies around 1 kHz and 100 kHz, suggesting the activation of two different charge transport channels. In the structure presented in Figure 2f, a similar phenomenon is observed at frequencies of 100 Hz and 1 MHz, indicating an even wider range of tunneling activity. This may result from greater microstructural variation in the material, for example, the presence of regions with different oxidation states or varying thicknesses of dielectric barriers between conductive grains. The presence of multiple tunneling mechanisms in these structures indicates the complex nature of electron transport in the tested nanocomposites and the significant effect of oxygen content on conductivity.

For higher temperatures, two characteristic frequency ranges can be distinguished in graphs (Figure 2a–c). In the low-frequency region < 10^4^ Hz, conductivity reaches a nearly constant value, corresponding to the direct current conductivity. However, for higher frequencies > 10^4^ Hz, a distinct increase in conductivity is observed. This behavior can be described using Jönscher’s law (Equation (3)).

(3) $$\sigma \left(f\right)=\sigma_{DC}+A\cdot f^{n}$$

where $\sigma_{DC}$—DC conductivity, A—material constant, n—exponent (0 < *n* < 1).

For the lowest temperatures < 172 K in samples made in 0%, 10%, and 20% of oxygen, an additional change in the slope of the curves is visible in the range of approximately 10^3^–10^4^ Hz. This means that a single power law does not correctly describe the entire course. In this case, it is necessary to use a double power law model. For the sample containing 30%, 40%, 50% of oxygen, the situation is different. The curves for temperatures of 20 K, 124 K, and 172 K practically coincide and form a single straight line across the entire frequency range. This means that in this case, a single power law is sufficient to describe the conductivity, suggesting the dominance of a single charge transport mechanism [56].

The dependence of AC conductivity on frequency was numerically analyzed using a power-law fit. For samples obtained in an oxygen atmosphere of 0–20% O_2_, a distinct dip is visible around 10^4^ Hz, particularly at higher temperatures [57]. Below this frequency, the DC component associated with long-range transport dominates, while above 10^4^ Hz, the conductivity increases according to Jönscher’s power law (Equation (4)).

(4) $$\sigma_{AC}=f^{n}$$

For higher oxygen contents of 30–50%, the dip disappears, and the log–log data form a straight line across the entire frequency range, indicating the dominance of the hopping mechanism and the homogenization of localization centers. Temperature analysis shows convergence of *σ* values for 0–20% O_2_ at high frequencies, while for 30% O_2_ at 375 K, a clear shift in conductivity by more than an order of magnitude toward higher values is observed.

The conductivity values obtained for the lowest applied frequency, corresponding to quasi-DC conditions, were analyzed over the entire temperature range from 20 to 375 K and for all oxygen contents tested. Good linearity of the analyzed relationship was obtained in the low-temperature range, indicating that the dominant charge transport mechanism in Ru–Si–O thin films is the VRH Mott hopping mechanism [52].

Analysis of the slopes of the lines approximating the individual temperature ranges allowed us to determine the activation energy corresponding to the jump of a single electron to the next potential well. These values reflect the energy required to overcome the potential barrier between adjacent charge centers. Higher activation energy values indicate stronger electron binding and larger barriers between potential levels, while lower values indicate easier charge transport and higher conductivity within a given temperature range. Similar situations have been observed in articles [12,58,59]. Experimentally determined activation energies for the Ru–Si–O system show a clear dependence on both the oxygen content and the measurement frequency. All obtained values are within the range of less than 1.5 meV, which is significantly lower than the thermal energy at room temperature. This indicates that the charge transport does not follow the classical thermally activated conduction pattern consistent with the Arrhenius model. Such low values indicate a jump-like conduction mechanism between localized states or tunneling transport, in which the effect of temperature is limited. At a frequency of 0.01 MHz, the dependence of E_a_ on oxygen content is nonmonotonic. The maximum activation energy was observed for the sample containing 20% of O_2_, where E_a_ increased almost 2.8-fold compared to the Ru–Si sample without added oxygen (from 0.44 meV to 1.22 meV). This indicates an increase in the number of localized states or a change in the percolation threshold, which limits the mobility of charge carriers. However, further increases in oxygen content lead to a lower activation energy. For 40% of O_2_, a value of approximately 0.19 meV was obtained. For the sample containing 50% of O_2_, a clear dependence of the activation energy on frequency was also observed. As the frequency increases from 0.001 MHz to 1.0 MHz, the E_a_ value decreases by approximately from 0.92 meV to 0.54 meV. This type of behavior is characteristic of AC hopping conduction and corresponds to the universal Jönscher dielectric response. Higher frequencies correspond to shorter transport distances, which allows carriers to jump between more closely spaced localized states and effectively overcome lower potential barriers [56].

As can be observed in Figure 3, at low temperatures (e.g., 151 K), the phase angle assumes negative values, approaching −90°, which is typical for ideal or conventional capacitors. In this range, the dominant process is charge storage at phase boundaries or in potential barrier regions, and the displacement current significantly exceeds the conduction current. This behavior indicates that at low temperatures, the samples behave as high-capacitance dielectrics, where energy losses are minimal and the resistive component can be neglected. With increasing temperature, especially at lower frequencies, the phase angle gradually increases, approaching a value of approximately 0°. This means that the system no longer behaves like an ideal capacitor and begins to exhibit properties characteristic of a parallel RC circuit, which simultaneously contains a resistive (conductive) and capacitive (energy-storing) component. This behavior can be interpreted as a result of the increase in conductivity with temperature, which leads to a significant contribution of conduction current to the total current flow through the material. At higher temperatures, the mobility of charge carriers increases, and potential barriers become easier to overcome, resulting in an increased contribution of resistive processes and a reduction in the phase shift angle. Ultimately, the observed transition from typical capacitor characteristics to RC behavior indicates a change in the dominant mechanism of electrical response with increasing temperature. At low temperatures, polarization and dielectric processes predominate, whereas at higher temperatures, charge conduction processes begin to play an increasingly important role in the behavior of the entire system.

On Figure 4 the minimum tanδ value at frequency from 10^3^ to 10^5^ Hz is the result of interfacial polarization (Maxwell–Wagner) and Debye relaxation which is described by Equation (5). Depending on the material’s composition and microstructure, this minimum shifts with frequency, which is associated with the characteristic relaxation time of the tested structure [58]. The dielectric loss tangent varies parabolically with frequency, which indicates a relaxation loss mechanism.

(5) $$tan\delta \;=\;1+\frac{\omega \tau }{1+\omega^{2}\tau^{2}}$$

where ω—angular frequency, τ—characteristic relaxation time.

Figure 5 shows the dependence of the frequency coefficient α as a function of frequency. In systems with different oxygen contents, the increase in the α a coefficient with frequency indicates the dominance of ionic conduction processes and the increasing role of interfacial barrier energy in the structure. However, the maximum value of the frequency coefficient alpha tends to decrease with increasing oxygen content during the preparation process. Furthermore, with increasing oxygen content, the maximum peak shifts towards lower frequencies. Two peaks (maximum values) can be clearly distinguished in the frequency coefficient α curve. The first appears in the frequency range of 10^2^–10^3^ Hz, while the second, with a much larger amplitude, is observed in the higher frequency range, i.e., 10^4^–10^6^ Hz. Comparing the shapes of the conductivity and frequency coefficient curves, presented in Figure 2 and Figure 5, respectively, reveals their significant similarity. This indicates that the observed relationships are consistent with a hopping charge transport mechanism, in which current carriers move between local energy states via the quantum phenomenon of electron tunneling. This consistency clearly indicates the presence of a hopping conduction mechanism in the tested nanocomposite. Furthermore, the occurrence of two distinct peaks suggests that at least two types of electron tunneling, differing in their characteristic relaxation times, operate within the material’s structure. This may be due to the existence of distinct conduction pathways. For different Ru-Si-O contents, shifts and changes in the peak intensity are observed, as well as a general trend of increasing alpha at higher frequencies, characteristic of materials with a predominance of nonlinear dispersion or Schottky barrier effects. The oxygen content influences the number and nature of transport-active sites, which translates into a change in conductivity dynamics and significant differences in the alpha coefficient value between the tested structures [59,60].

(6) $$\alpha =\frac{\log\sigma_{i+1}-\log\sigma_{1}}{\log f_{i+1}-\log f_{1}}$$

where i—measuring point number.

Dependencies in graphs (Figure 6) indicates the occurrence of a relaxation process associated with Maxwell–Wagner–Sillars interfacial polarization, occurring at the interface between conducting nanoparticles and an insulating matrix. In this mechanism, electric charges accumulate at the interface of two phases with different conductivities, leading to a delayed response of the system to an applied electric field [61]. Analysis of the imaginary component εʺ confirms this type of relaxation. At low frequencies, εʺ values are very high, indicating a significant contribution of conduction losses, primarily resulting from the motion of charge carriers in the dielectric medium containing dispersed nanoparticles. Above approximately 1 kHz, a distinct decrease in εʺ values is observed, indicating a gradual reduction in conduction losses with increasing frequency. In this range, ionic transport processes become less important, and the material response is increasingly related to structural and orientational polarization [62].

### 4.2. Analysis of the Chemical Composition of Ru-Si-O Structures

A clear compositional evolution with increasing oxygen content in the sputtering atmosphere is observed, reflecting the progressive incorporation of oxygen into the Ru–Si system during reactive sputtering. At 0% O_2_, the film exhibits a high Ru content (24.9 at.%) and moderate Si (50.2 at.%), with only a minor oxygen contribution (24.9 at.%). This composition corresponds to a metallic Ru–Si alloy, consistent with the absence of oxygen in the plasma. Such films are expected to exhibit metallic conductivity, which is in line with the electrical behavior observed for this series. With the introduction of oxygen ranging from 10 to 30%, a pronounced increase in oxygen content and a concomitant decrease in Ru concentration are observed. At 10% of O_2_, the atomic oxygen fraction rises sharply to approximately 78 at.%, while Ru decreases to about 3 at.%. This trend continues up to 30% of O_2_, where Ru falls below 2 at.%. These results clearly indicate the transition from metallic Ru–Si to a mixed oxide regime dominated typically by SiO_2_ and RuO_2_ phases. While the absolute values, particularly for oxygen, should be interpreted with caution due to the well-known limitations of EDS for light-element quantification, these results clearly demonstrate a transition from a Ru–Si-rich regime toward an oxygen-rich matrix. The observed compositional trends are consistent with the progressive oxidation of the system, where silicon preferentially forms an amorphous SiOₓ network, and ruthenium becomes increasingly diluted within the growing oxide matrix and/or partially oxidized. The decreasing Ru signal intensity may therefore reflect a combination of Ru oxidation, redistribution within the amorphous oxide matrix, and the reduced sensitivity of EDS at very low Ru concentrations. At oxygen fractions of 40% and 50%, Ru becomes close to the detection limit of EDS, with measured values of 1.3 at.% and 0.7 at.%, respectively. In this regime, the films are dominated by Si and O, suggesting a predominantly dielectric matrix. Although the presence of oxidized Ru species cannot be conclusively established by EDS alone, the compositional evolution and electrical behavior are consistent with an amorphous SiO_2_-rich matrix containing only trace amounts of Ru-based species. A definitive determination of chemical bonding and oxidation states would require complementary techniques such as XPS or WDS.

Analyzing Figure 8a and the electrical characteristics presented in Figure 2, it can be concluded that an increase in the relative Si-to-O content is associated with a decrease in electrical conductivity. This relationship is evident when comparing the oxygen-free film with the film deposited at 50% oxygen content, supporting the conclusion that the progressive formation of an oxygen-rich SiOₓ matrix governs the electrical transport properties of the Ru–Si–O films.

The diffractograms (Figure 9) show amorphous structures, with a peak at 21.3° corresponding to the quartz substrate. For Ru-Si thin film deposited without oxygen in the reactor chamber, we observe a broad line around 43°, which most likely comes from Ru (002) or Ru_2_Si_3_ (222) crystalline inclusions within an amorphous matrix, as can be inferred from previous structural studies published in [24,44].

## 5. Conclusions

Analysis of the frequency- and temperature-dependent conductivity revealed that charge transport in the studied materials is dominated by a hopping conduction mechanism (conduction by electron hopping) between localized energy states. This mechanism, characterized by thermally activated conduction, confirms that thermal energy facilitates electron hopping between localized states, leading to an increase in the sample’s overall conductivity with increasing temperature. In samples with 30% and 50% oxygen content, the presence of more than one electron hopping mechanism, characterized by different activation energies, was observed. This phenomenon indicates a complex microscopic structure of the material, with the possibility of regions with different oxidation states and variable dielectric barrier thicknesses between the conductive grains. Exceptional behavior was observed in the oxygen-free sample (0%), where a decrease in conductivity characteristic of metallic conduction was observed up to a temperature of approximately 172 K. Above this temperature, a transition in the nature of conduction occurred. Conductivity began to increase with temperature, indicating a shift from a metallic to a dielectric mechanism. The Maxwell–Wagner interface polarization observed in the structures results from charge accumulation at the interface between the conductive nanoparticles and the insulating matrix. The minimum of the dielectric loss tangent observed in the frequency range of 10^3^–10^5^ Hz is the result of Debye relaxation and polarization phenomena. The parabolic dependence of the loss tangent on frequency indicates a relaxation-related loss mechanism, and the change in this minimum with frequency is related to the characteristic relaxation time of the studied structure. Among the tested samples, those containing oxygen in amounts ranging from 0 to 20% exhibited the best capacitive parameters, which is related to the change in phase angle from 0° to −90°. Taking this into account, the obtained samples can be used as parallel-connected RC systems.

In summary, chemical composition results confirm that increasing the oxygen fraction in the sputtering atmosphere from 0 to 50% leads to a systematic oxidation of the Ru–Si system, transforming the film composition from metallic Ru–Si to a predominantly SiO_2_-based structure with trace RuO_2_ content. The observed trends are consistent with the expected transition from metallic to dielectric behavior, which is further supported by the corresponding changes in the electrical and dielectric properties of the films.

The key finding of the study is that the conductivity value does not depend directly on the amount of oxygen used during sample preparation, but rather on the actual silicon content in the material structure, when its relative oxygen content exceeds this value. This is confirmed by energy-dispersive X-ray spectroscopy (EDS) analyses, which demonstrated that the silicon atomic content is a key parameter governing electron transport properties. Increasing the oxygen content leads to an increase in the number of dielectric barriers between the ruthenium nanoparticles and the silicon phases, which affects the conductivity dynamics and the differences in conduction mechanisms between the studied structures.

Based on the analysis of the existing literature and the results obtained in this article, it can be concluded that the obtained structures demonstrate promising application properties. A comparison of previous reports with the conducted research indicates that these materials possess properties that are important for practical use. Therefore, it can be concluded that they have potential applications in both biomedical and environmental engineering, particularly in areas requiring advanced, functional material solutions.

## Figures and Tables

**Table 1:** The value of the electron hopping activation energy in samples with different oxygen content.

Material	Oxygen Content [%]	Frequency [MHz]	Activation Energy [eV]
Ru–Si	0	0.01	(4.4 ± 1.97)∙10^−4^
Ru–Si–O	10	0.01	(8.16 ± 1.42)∙10^−4^
	20	0.01	1.22∙10^−3^ ± 1.42∙10^−4^
	30	0.01	(8.36 ± 2.13)∙10^−4^
	40	0.01	(1.85 ± 3.94)∙10^−4^
	50	0.001 1	(9.15 ± 2.05)∙10^−4^ (5.43 ± 1.17)∙10^−4^

## Data Availability

The original contributions presented in this study are included in the article. Further inquiries can be directed to the corresponding author.
